# Supplementary material for: Protein Phosphatase 4 Is Required for Centrobin Function in DNA Damage Repair
Source: Cells. 2023 Sep 6;12(18):2219. doi: 10.3390/cells12182219 (PMC10526779; doi:10.3390/cells12182219)

Supplementary Figures

Protein Phosphatase 4 is Required for Centrobin Function in DNA Damage Repair

Zsuzsánna Réthi-Nagy [1,2], Edit Ábrahám [1,3], Rita Sinka [4], Szilvia Juhász [5,*] and Zoltán Lipinszki [1,3,*]

[1] MTA SZBK Lendület Laboratory of Cell Cycle Regulation, Institute of Biochemistry, HUN-REN Biological Research Centre, H-6726 Szeged, Hungary; nagy.zsuzsanna@brc.hu (Z.R.-N.); abraham.edit@brc.hu (E.Á.)

[2] Doctoral School of Biology, Faculty of Science and Informatics, University of Szeged, H-6726 Szeged, Hungary

[3] National Laboratory for Biotechnology, Institute of Genetics, HUN-REN Biological Research Centre, H-6726 Szeged, Hungary

[4] Department of Genetics, University of Szeged, H-6726 Szeged, Hungary; rsinka@bio.u-szeged.hu

[5] Institute of Biochemistry, HUN-REN Biological Research Centre, H-6726 Szeged, Hungary

* Correspondence: juhasz.szilvia@brc.hu, lipinszki.zoltan@brc.hu

Figure S1

MATSADSPSSPLGAEDLLSDSSEPPGLNQVSSEVTSQLYASLRLSRQAEATARAQLYLPSTSPPHE
GLDGFAQELSRSLSVGLEKNLKKKDGSKHIFEMESVRGQLQTMLQTSRDTAYRDPLIPGAGSER
REEDSFDSDSTATLLNTRPLQDLSPSSSAQALEELFPRYTSLRPGPPLNP PDFQGLRDALDSEHT
RRKHCERHIQSLQTRVLELQQQLAVAVAADRKKDTMIEQLDKTLARVVEGWNRHEAERTEVL
RGLQEEHQAAELTRSKQQETVTRLEQSLSEAMEALNREQESARLQQRERETLEEERQALTLRLE
AEQQRCCVLQEERDAARAGQLSEHRELETLRAALEEERQTWAQQEHQLKEHYQALQEESQA
QLEREKEKSQREAQAAWETQHQLALVQSEVRRLEGELDTARRERDALQLEMSLVQARYESQRI
QLESELAVQLEQRV TERLAQAQESSLRQAASLREHHRKQLQDLSGQHQQELASQLAQFKV
EMAEREERQQQVAEDYELRLAREQARVCELQSGNQQLEEQRVELVERLQAMLQAHWDEAN
QLLSTTLPPPNPPAPPAGPSSPGPQEPEKEERRVWTMPPMAVALKPVLQQSREARDELPGAPP
VLCSSSSDLSLLLGPSFQSQHSFQPLEPKPDLTSSTAGAFSALGAFHPDHRAERPFPEEDPGPDGE
GLLKQGLPPAQLEGLKNFLHQLLETVPQNNENPSVDLLPPKSGPLTVPSWEEAPQVPRIPPPVHK
TKVPLAMASSLFRVPEPPSSHSQGSGPSSGSPERGGDGLTFPRQLMEVSQLLRLYQARGWGAL
PAEDLLLYLKRLEHSGTDGRGDNVPRRNTDSRLGEIPRKEIPSQAVPRRLATAPKTEKPPARKKSG
HPAPSSMRSRGGVWR

(a)

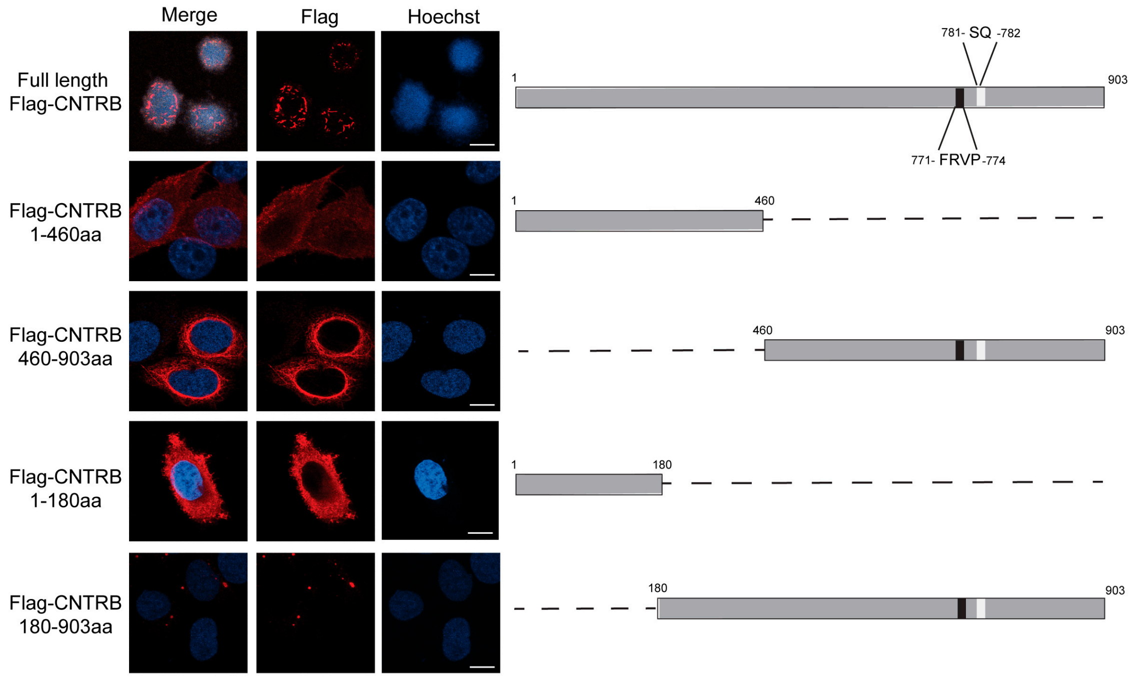

(b)

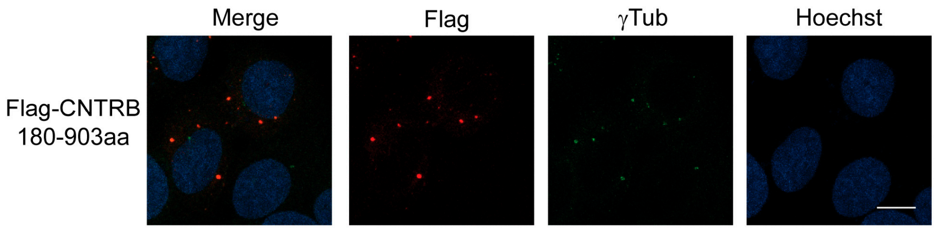

(c)

Figure S1. The localization of the various CNTRB fragments in human cells. (**a**) The secondary structure of CNTRB was predicted with PsiPred (http://bioinf.cs.ucl.ac.uk/psipred/). The endpoints of the generated fragments are indicated by red squares. Alpha-helical regions are denoted in bold and underlined. The FxxP (FRVP, highlighted in grey) motif serves as

the binding site for R3 subunits, while SQ (in red) is a putative phosphorylation site targeted by ATR kinase. (**b**). Left panel: Representative images of expression of full length and truncated forms of Flag-tagged CNTRB. Cells were transfected with CNTRB fragments-encoding plasmid construct, and 48 h post-transfection cells were fixed and stained with anti-FlagM2 (red) and Hoechst (DNA, blue). Scale bar: 10 μm. Right panel: The schematic representation of truncated forms of CNTRB. The binding motif for R3 subunits, FRVP, and the putative phosphorylation site for ATR kinase, SQ, are indicated. Numbers indicate amino acid endpoints. (**c**). Representative images of Flag-CNTRB$^{180\text{-}903aa}$ fragment (red) co-localize with the centrosomal marker γ-Tub (green). Scale bar: 10 μm.

Figure S2

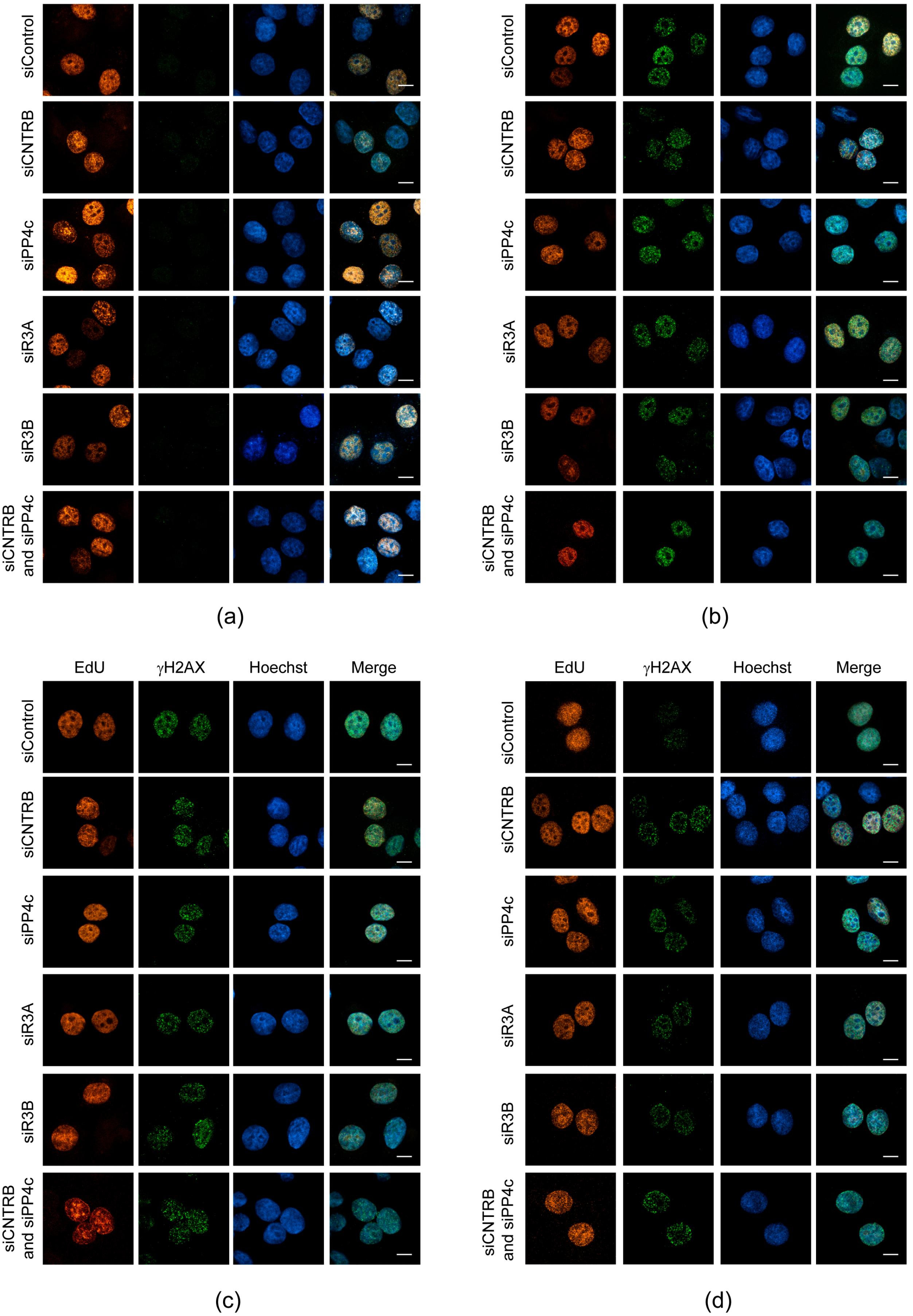
siControl
siCNTRB
siPP4c
siR3A
siR3B
siCNTRB and siPP4c
(a)
(b)
EdU
γH2AX
Hoechst
Merge
(c)
(d)

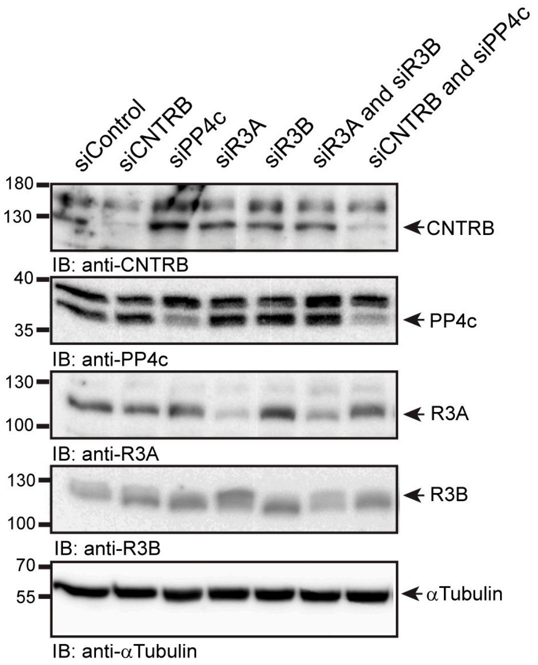

siControl
siCNTRB
siPP4c
siR3A
siR3B
siR3A and siR3B
siCNTRB and siPP4c
180
130
CNTRB
IB: anti-CNTRB
40
35
PP4c
IB: anti-PP4c
130
100
R3A
IB: anti-R3A
130
100
R3B
IB: anti-R3B
70
55
αTubulin
IB: anti-αTubulin

(e)

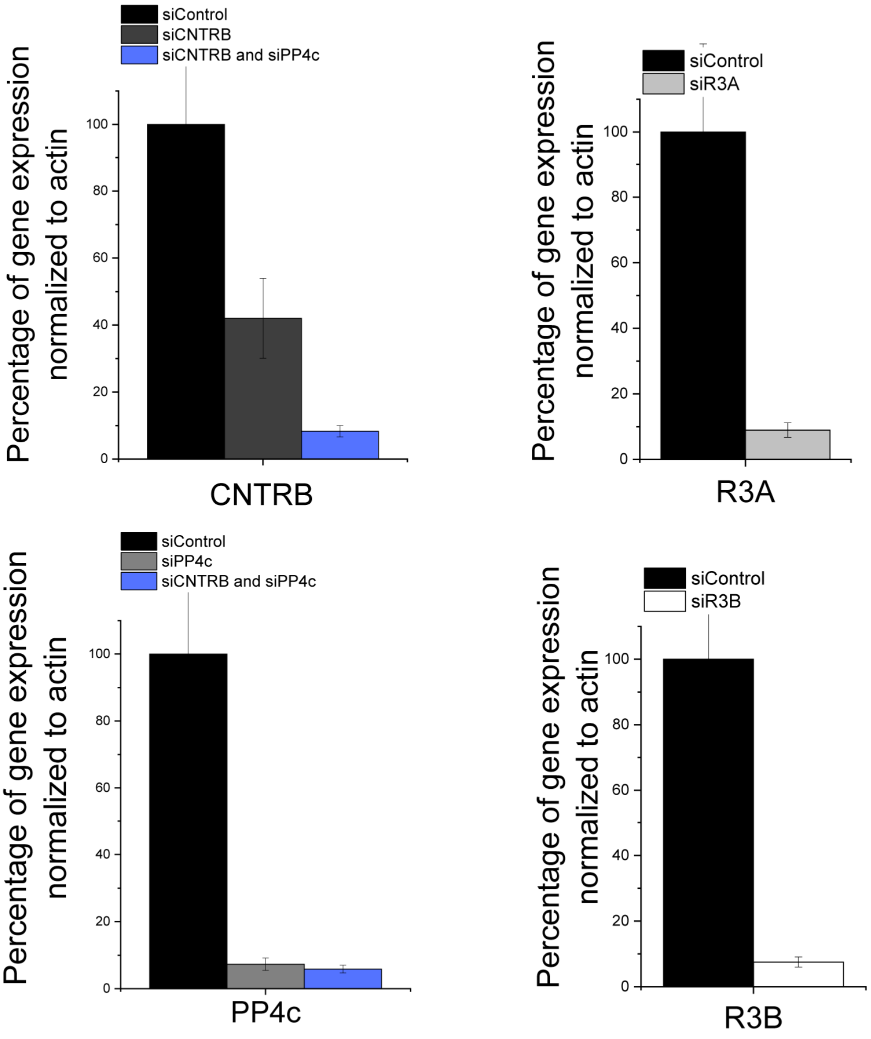

siControl
siCNTRB
siCNTRB and siPP4c
Percentage of gene expression normalized to actin
CNTRB
siControl
siR3A
R3A
siControl
siPP4c
siCNTRB and siPP4c
PP4c
siControl
siR3B
R3B

(f)

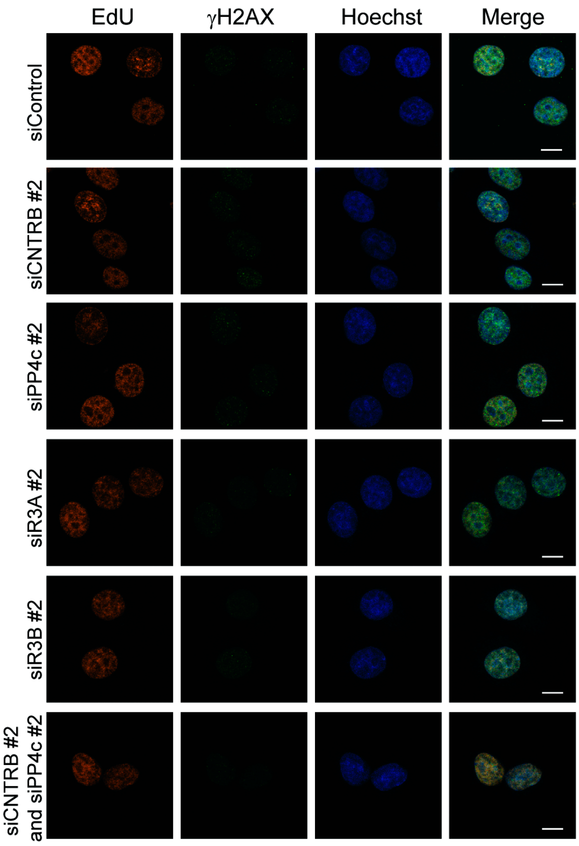
EdU
γH2AX
Hoechst
Merge
siControl
siCNTRB #2
siPP4c #2
siR3A #2
siR3B #2
siCNTRB #2 and siPP4c #2

(g)

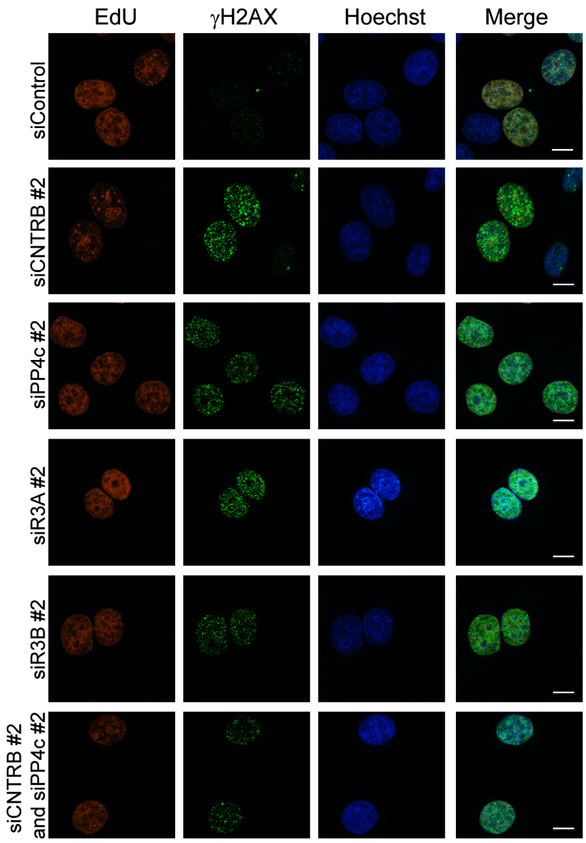
EdU
γH2AX
Hoechst
Merge
siControl
siCNTRB #2
siPP4c #2
siR3A #2
siR3B #2
siCNTRB #2 and siPP4c #2

(h)

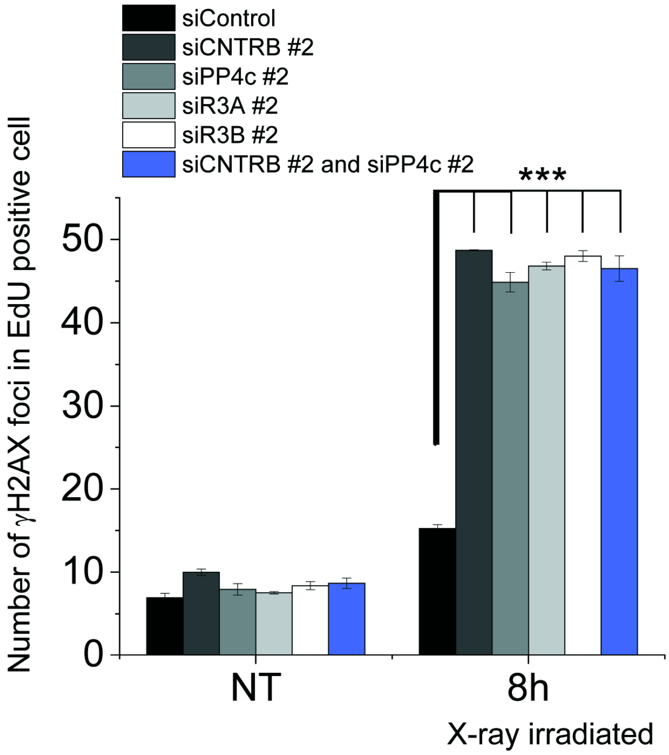
siControl
siCNTRB #2
siPP4c #2
siR3A #2
siR3B #2
siCNTRB #2 and siPP4c #2
***
Number of γH2AX foci in EdU positive cell
0
10
20
30
40
50
NT
8h
X-ray irradiated

(i)

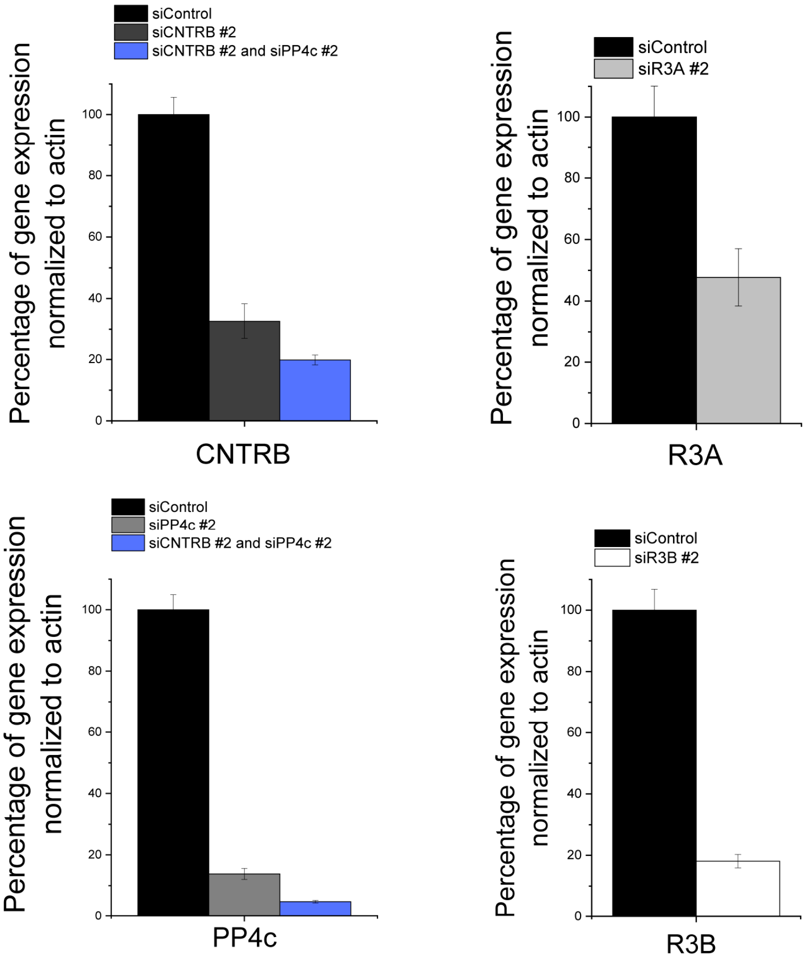
siControl
siCNTRB #2
siCNTRB #2 and siPP4c #2
Percentage of gene expression normalized to actin
CNTRB
siControl
siR3A #2
Percentage of gene expression normalized to actin
R3A
siControl
siPP4c #2
siCNTRB #2 and siPP4c #2
Percentage of gene expression normalized to actin
PP4c
siControl
siR3B #2
Percentage of gene expression normalized to actin
R3B
0
20
40
60
80
100

(j)

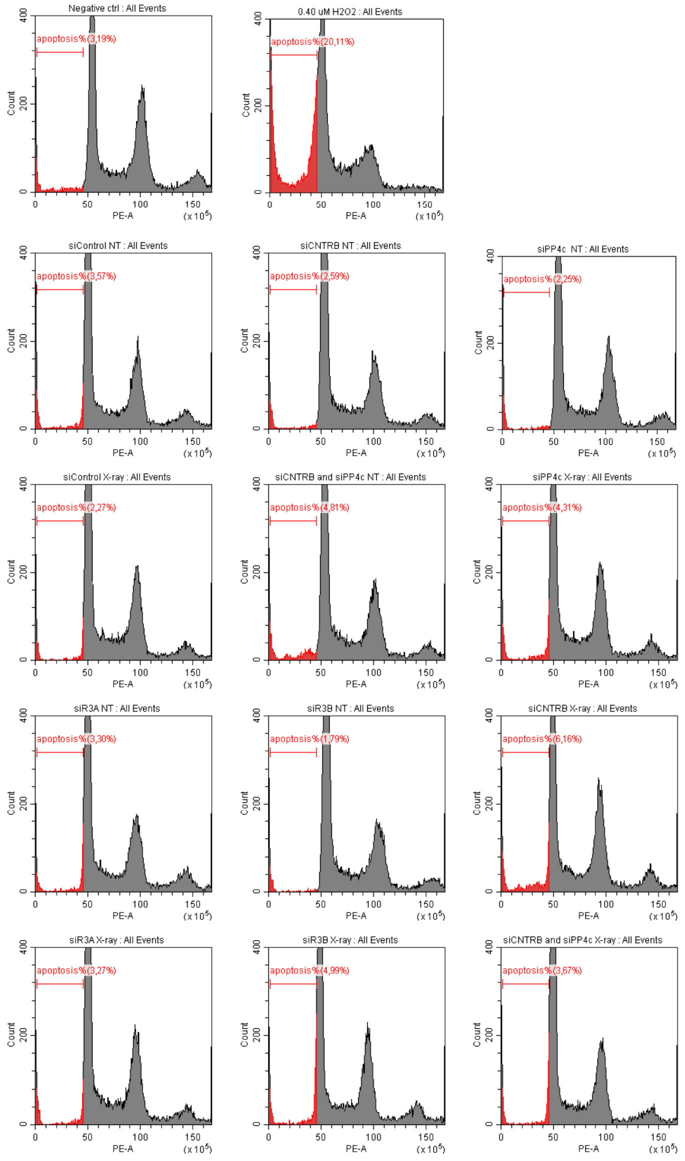

(k)

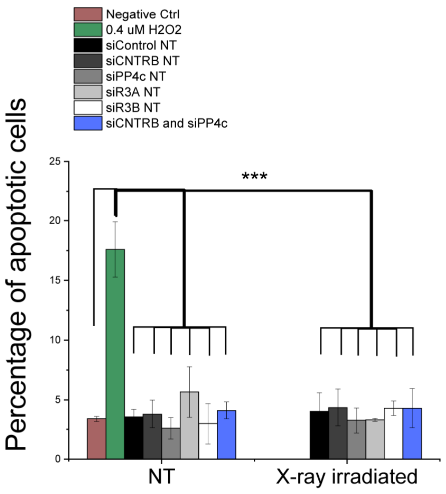

(l)

Figure S2. PP4 and CNTRB act together during DNA damage response. (**a-d**) Quantification of γH2AX in HeLa cells. Cells were transfected with the following siRNAs (Set #1): siControl, siCNTRB, siPP4c, siR3A, or siR3B, and siCNTRB and siPP4c together. 48 h post-transfection the damage was induced with X-ray irradiation (2 Gy). Microscopy images represent the γH2AX foci (green) in non-treated (Panel a) or in X-ray irradiated S phase cells (EdU, orange) 2h (Panel b), 4h (Panel c) and 8 h (Panel d) post-irradiation. Hoechst staining shows DNA (blue). Scale bar: 10 μm. (**e-f**) Validation of gene silencing by western blotting (Panel e) and qPCR (Panel f). 48 h post-transfection with the indicated siRNAs, cells were harvested and protein levels of CNTRB, PP4c, R3A or R3B were analyzed by western blotting using specific antibodies as indicated on the right. αTubulin serves as a loading control. In parallel, silencing of *cntrob*, *pp4c*, *r3a* or *r3b* was measured by qPCR. The values were normalized to the level of actin. (**g-i**) Quantification of γH2AX in HeLa cells. Cells were transfected with the second set of the following siRNAs (set #2): siControl, siCNTRB, siPP4c, siR3A, siR3B, and siCNTRB and siPP4c together. 48 h post-transfection DNA damage was induced with X-ray irradiation (2 Gy). Microscopy images represent the γH2AX foci (green) in non-treated (Panel g) or X-ray irradiated S phase (EdU, orange) cells 8 h (Panel h) post-irradiation. Hoechst staining shows DNA (blue). Scale bar: 10 μm. (**i**) A total of 50 cells after RNAi (set #2 siRNA) were examined at

various time points after irradiation to count the number of γH2Ax foci. The term "NT" refers to non-treated (non-irradiated) cells. The graphs display all data points along with the mean value and standard error of the mean (SEM) (n = 3). Asterisks on the graphs represent the P values obtained through linear regression, which were calculated independently for each time point. (**j**) Validation of gene silencing by qPCR. 48 h post-transfection cells were harvested and the level of gene expression of silenced *cntrob*, *pp4c*, *r3a* or *r3b* with the second set of siRNAs was measured by qPCR. The values were normalized to the level of actin**.** (**k**) The calculation of the proportion of intact and apoptotic nuclei by flow cytometry analysis. 0.4 μM H_2O_2-treatment was used as a positive control for apoptosis induction. (l) The assessment of DNA content in cells after RNAi involved the investigation of the apoptotic cell percentage following irradiation. The designation "NT" pertains to cells that underwent no treatment (i.e., were not irradiated). The graphs portray comprehensive data points in conjunction with the mean value and the standard error of the mean (SEM) (n = 3). The presence of asterisks on the graphs signifies the P values resulting from separate linear regression calculations conducted for each time point.

Figure S3

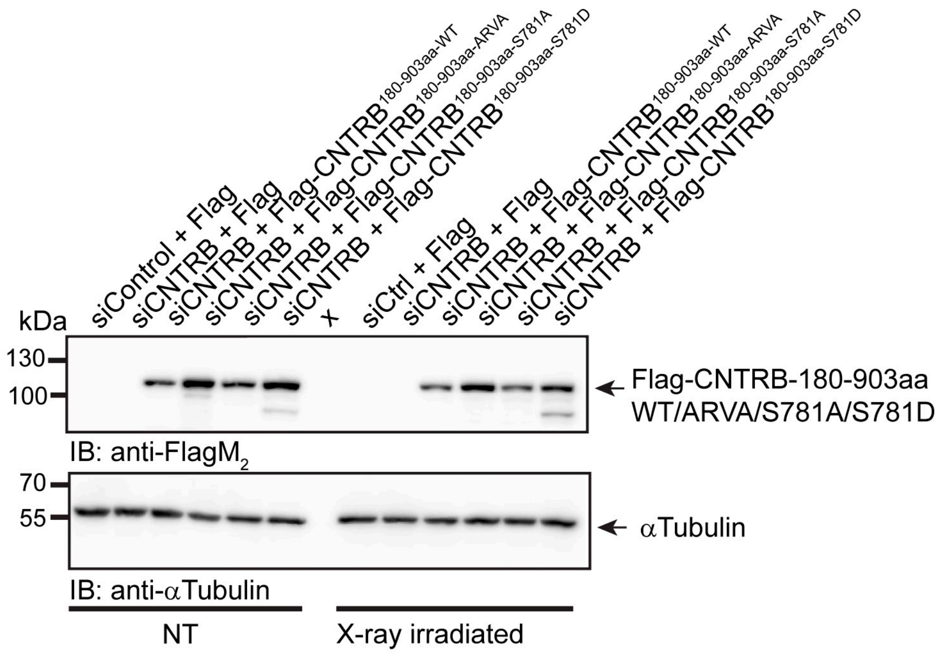

siControl + Flag
siCNTRB + Flag
siCNTRB + Flag-CNTRB180-903aa-WT
siCNTRB + Flag-CNTRB180-903aa-ARVA
siCNTRB + Flag-CNTRB180-903aa-S781A
siCNTRB + Flag-CNTRB180-903aa-S781D
x
siCtrl + Flag
siCNTRB + Flag
siCNTRB + Flag-CNTRB180-903aa-WT
siCNTRB + Flag-CNTRB180-903aa-ARVA
siCNTRB + Flag-CNTRB180-903aa-S781A
siCNTRB + Flag-CNTRB180-903aa-S781D
kDa
130
100
Flag-CNTRB-180-903aa
WT/ARVA/S781A/S781D
IB: anti-FlagM2
70
55
αTubulin
IB: anti-αTubulin
NT
X-ray irradiated

(a)

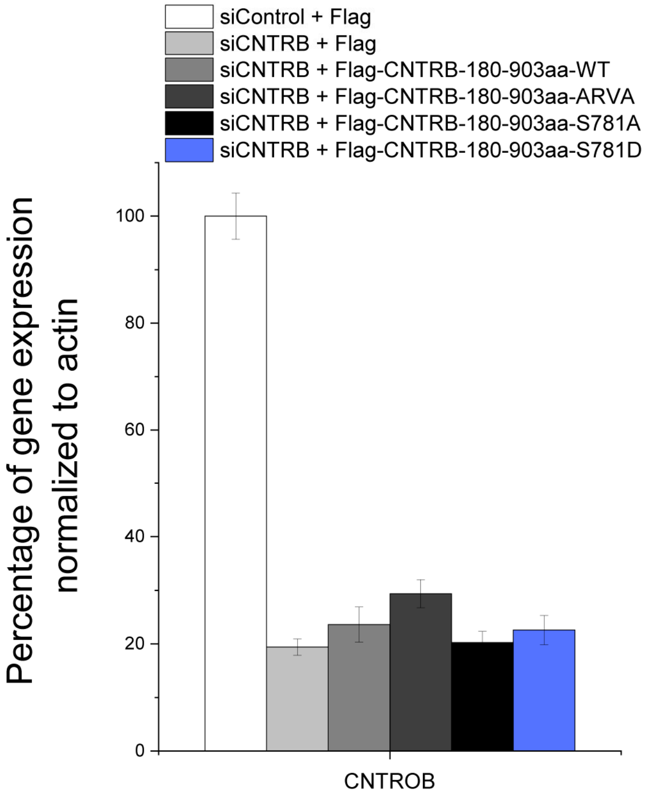

siControl + Flag
siCNTRB + Flag
siCNTRB + Flag-CNTRB-180-903aa-WT
siCNTRB + Flag-CNTRB-180-903aa-ARVA
siCNTRB + Flag-CNTRB-180-903aa-S781A
siCNTRB + Flag-CNTRB-180-903aa-S781D
Percentage of gene expression normalized to actin
0
20
40
60
80
100
CNTROB

(b)

Figure S3. The FRVP and SQ motifs are crucial for CNTRB-regulated DNA repair. (**a**) The expression level of wild-type or S781A/S781D/ARVA mutant variants of Flag-tagged CNTRB180-903aa in control or CNTRB-depleted cells was tested by Western blotting using the indicated antibodies. This shows comparable levels of the overexpressed Flag-tagged transgenic proteins. (**b**) Validation of gene silencing by qPCR. 48 h post-transfection cells were harvested and the level of gene expression of silenced CNTRB was measured by qPCR. The values were normalized to the level of actin.

Figure S4

Uncropped images corresponding to the main and supplementary figures (as indicated).

Figure 1b (Uncropped)

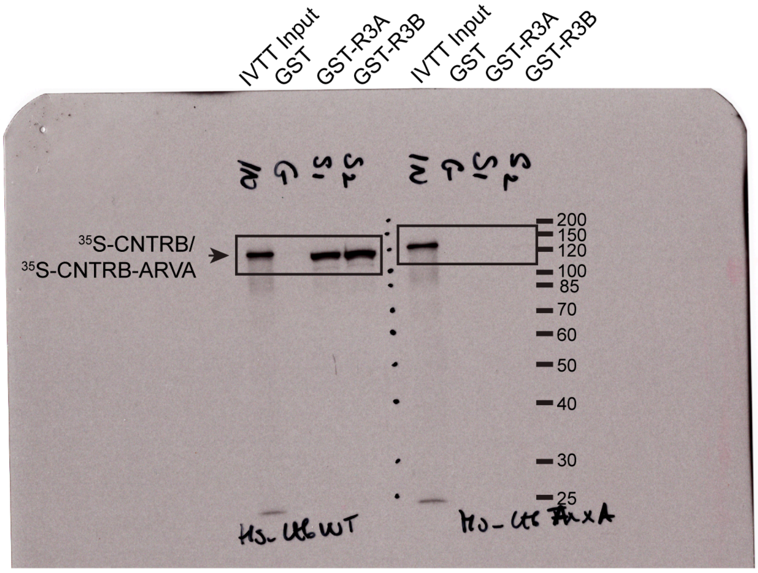

Figure 1c (Uncropped)

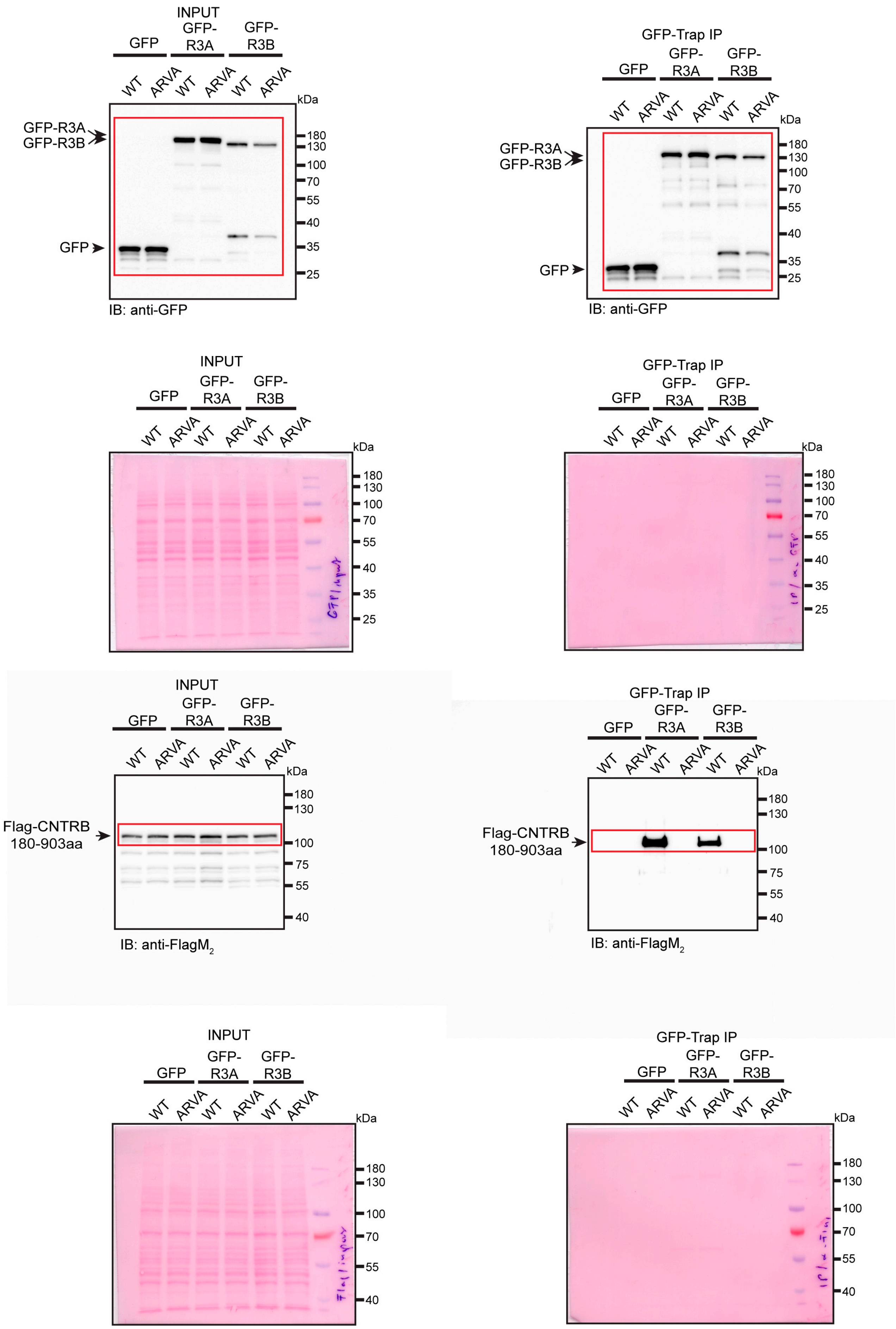

Figure 2d (Uncropped)

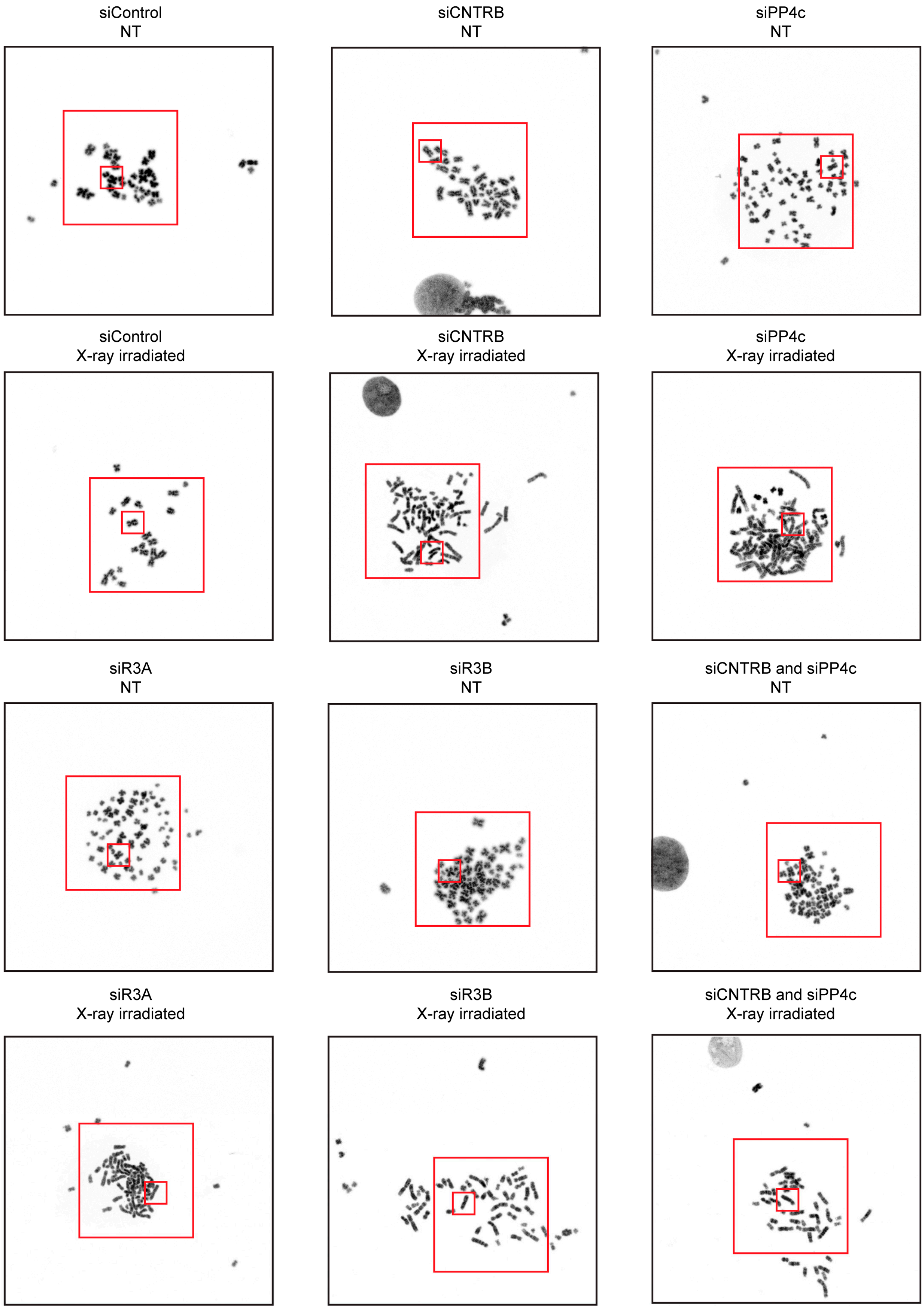

Figure 3a (Uncropped)

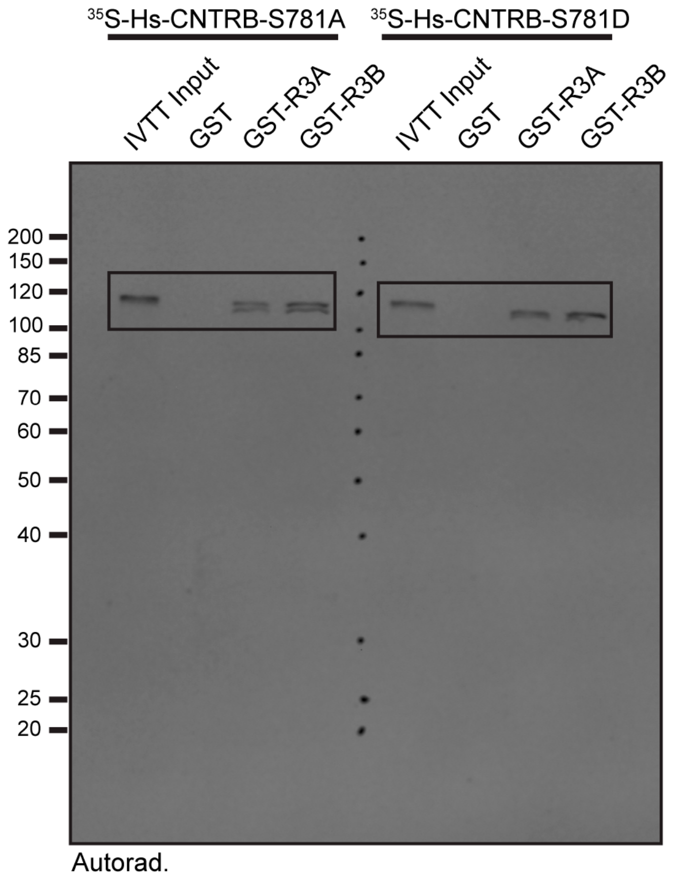

Figure 3b (Uncropped)

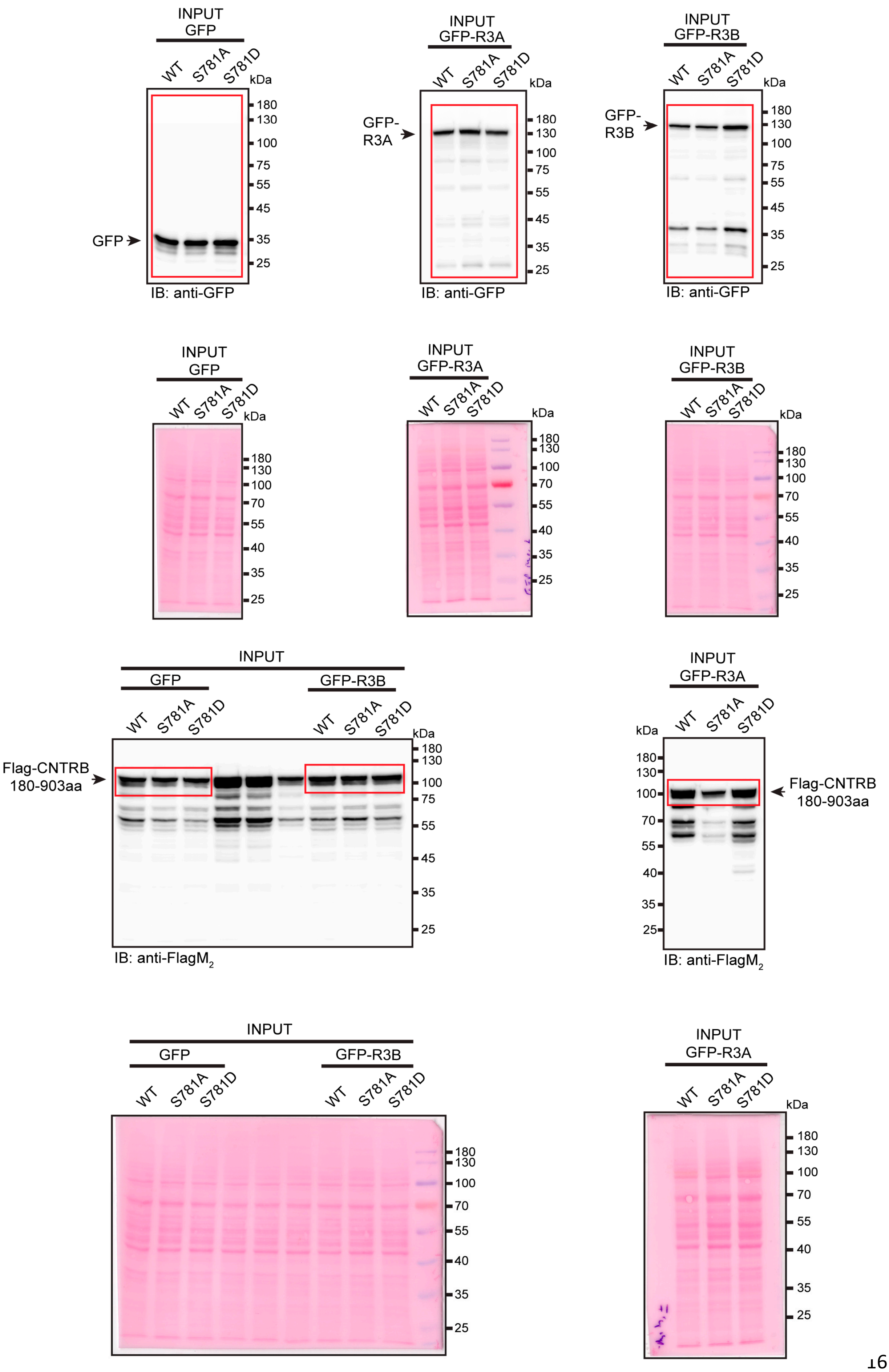

Figure 3b (Uncropped)

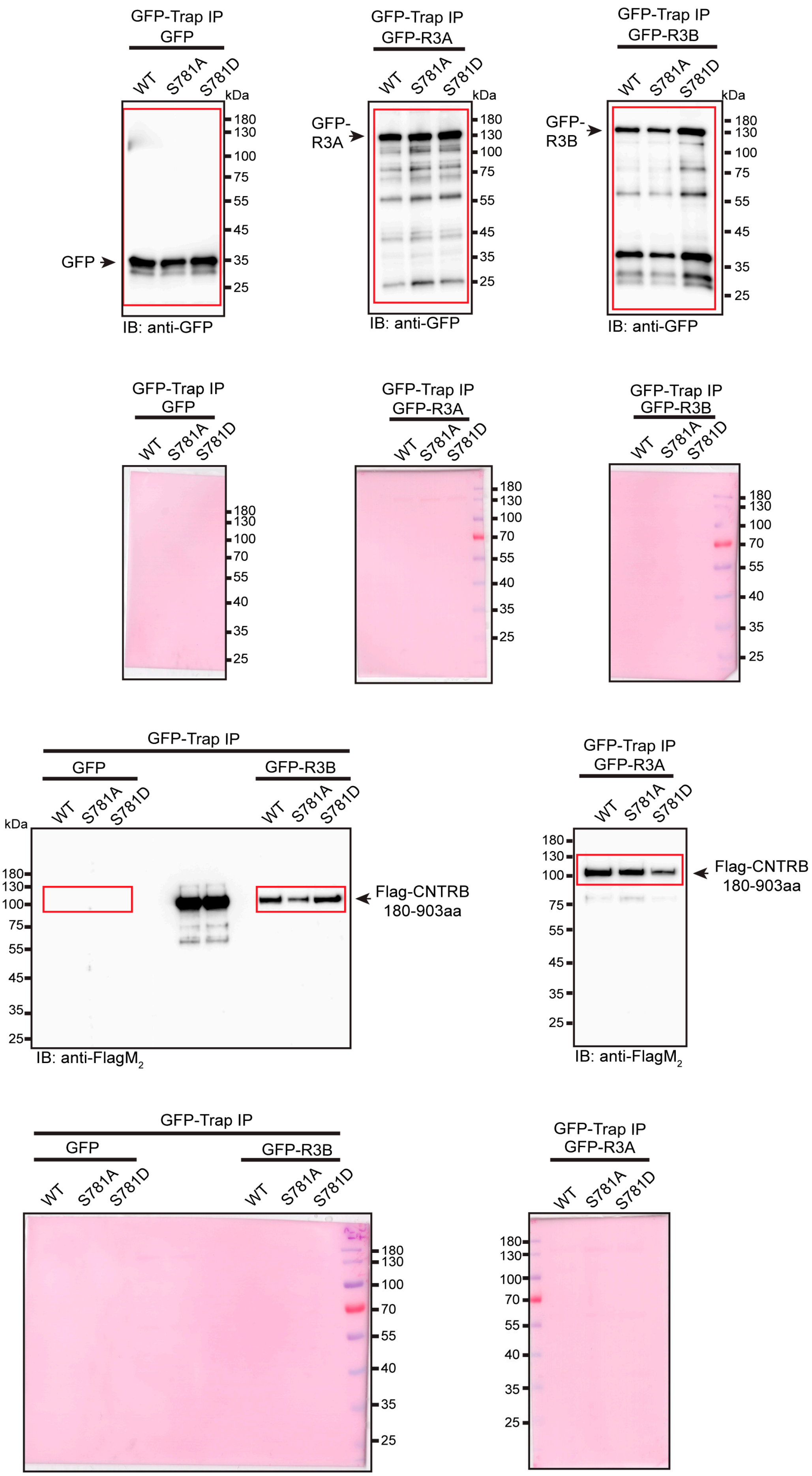

Figure 3d (Uncropped)

siControl
Flag
NT

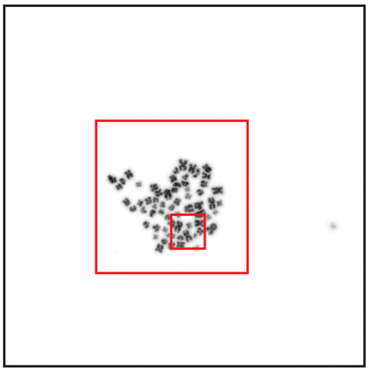

siCNTRB
Flag
NT

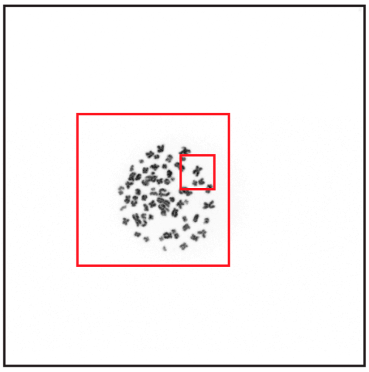

siCNTRB
Flag-CNTRB-180-903aa-WT
NT

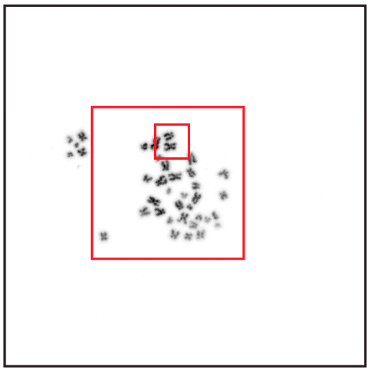

siControl
Flag
X-ray irradiated

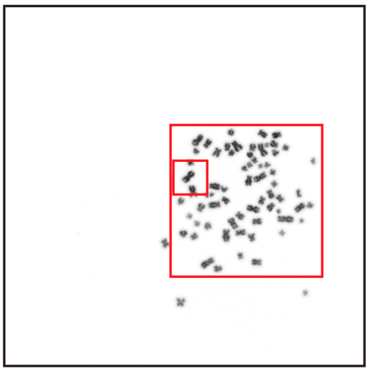

siCNTRB
Flag
X-ray irradiated

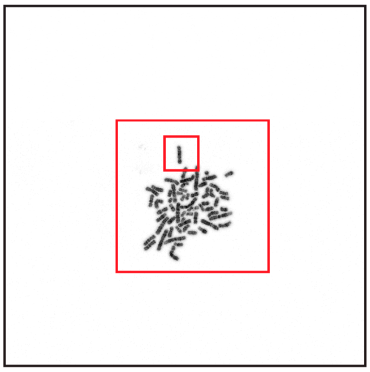

siCNTRB
Flag-CNTRB-180-903aa-WT
X-ray irradiated

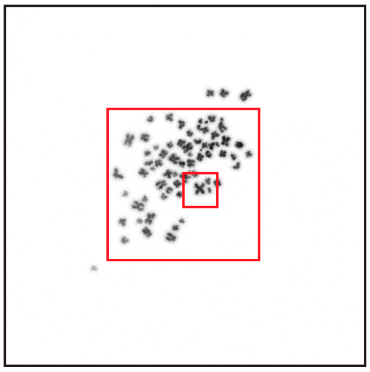

siCNTRB
Flag-CNTRB-180-903aa-ARVA
NT

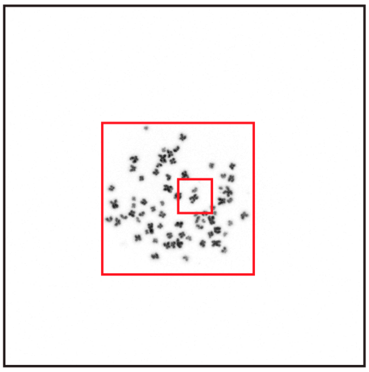

siCNTRB
Flag-CNTRB-180-903aa-S781A
NT

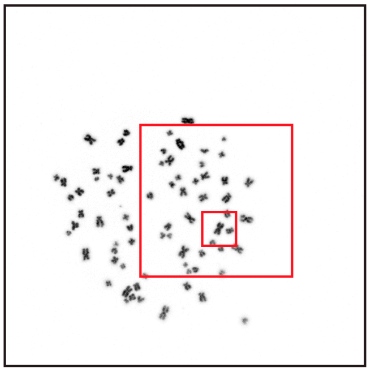

siCNTRB
Flag-CNTRB-180-903aa-S781D
NT

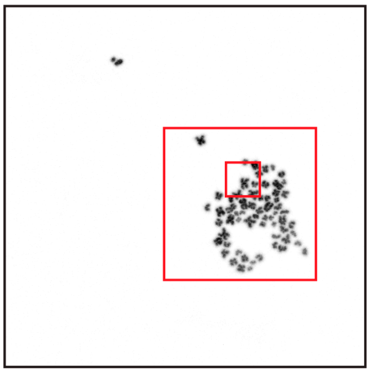

siCNTRB
Flag-CNTRB-180-903aa-ARVA
X-ray irradiated

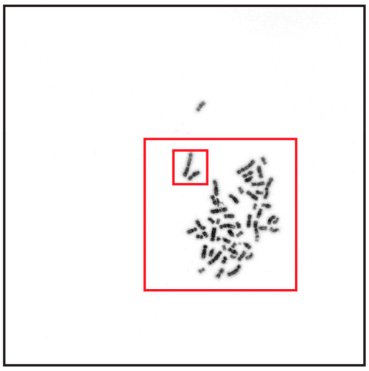

siCNTRB
Flag-CNTRB-180-903aa-S781A
X-ray irradiated

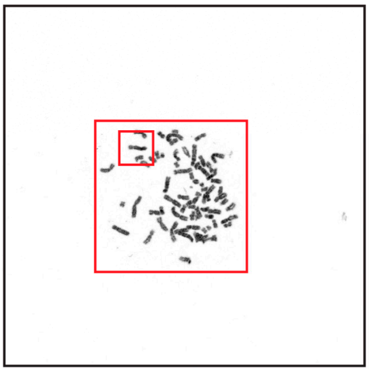

siCNTRB
Flag-CNTRB-180-903aa-S781D
X-ray irradiated

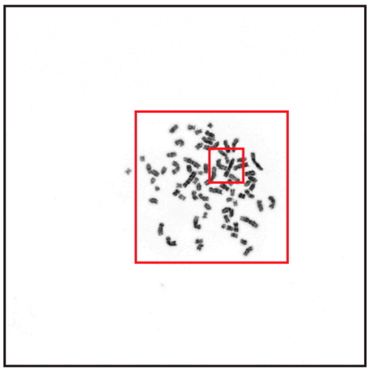

Supplementary figure 1b (Uncropped)

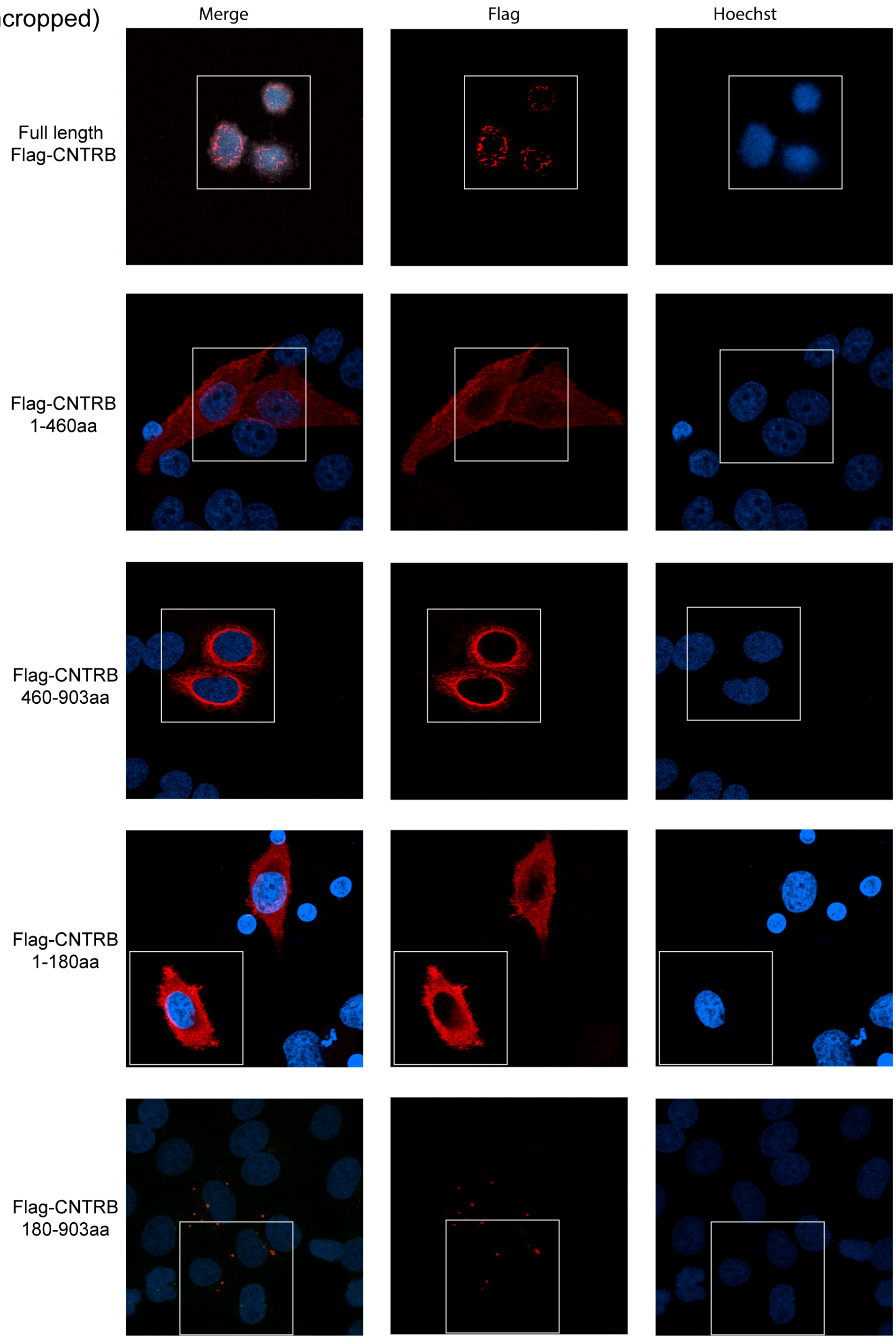

Supplementary figure 1c (Uncropped)

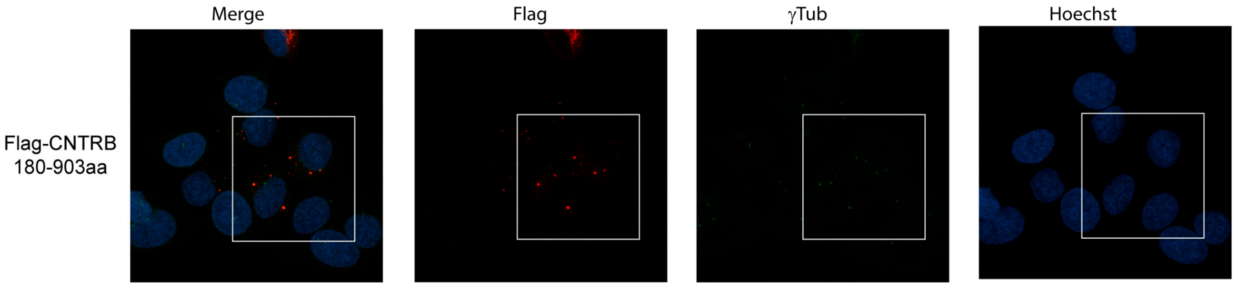

Supplementary figure 2e (Uncropped)

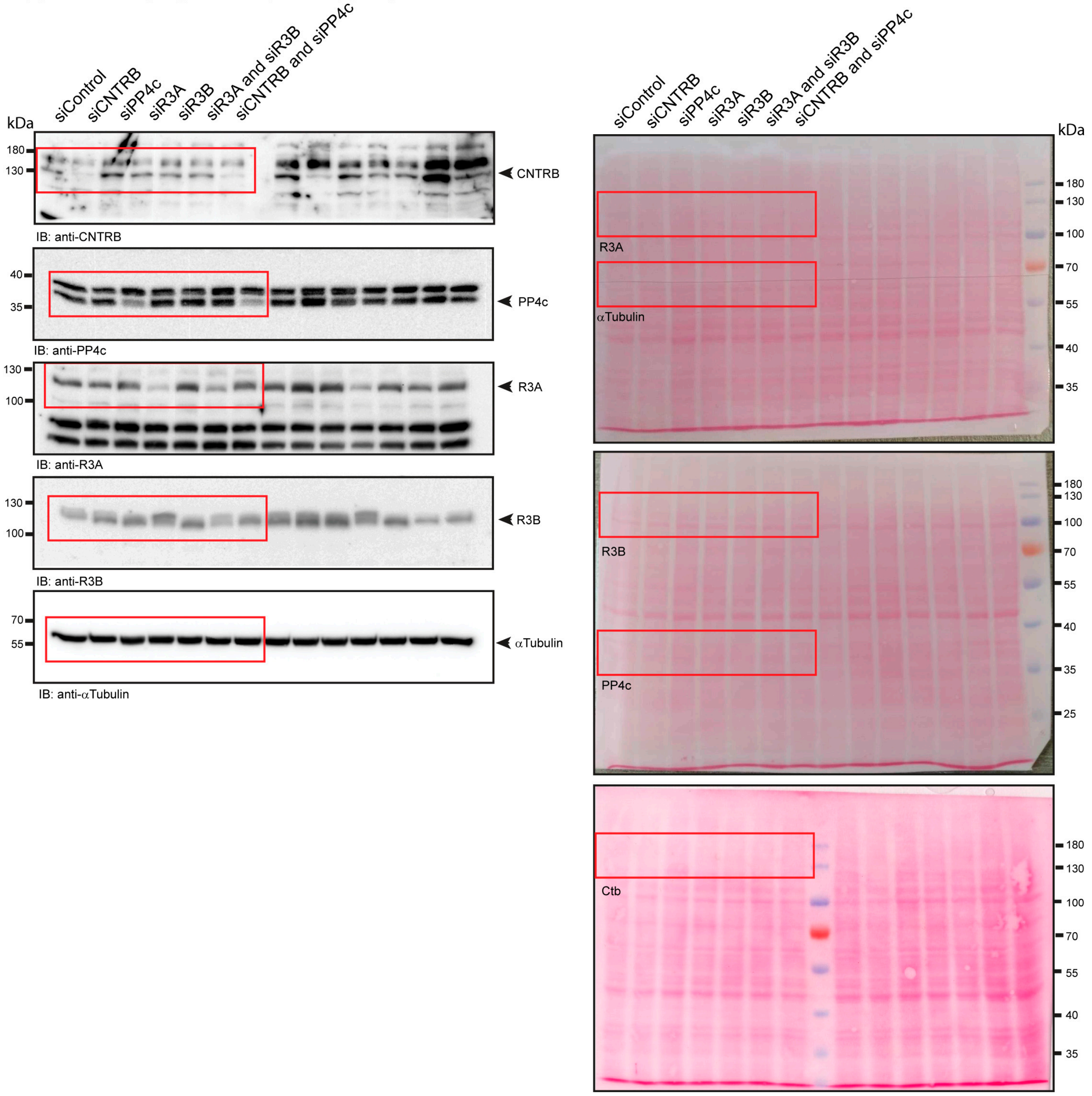

Supplementary figure 3a (Uncropped)

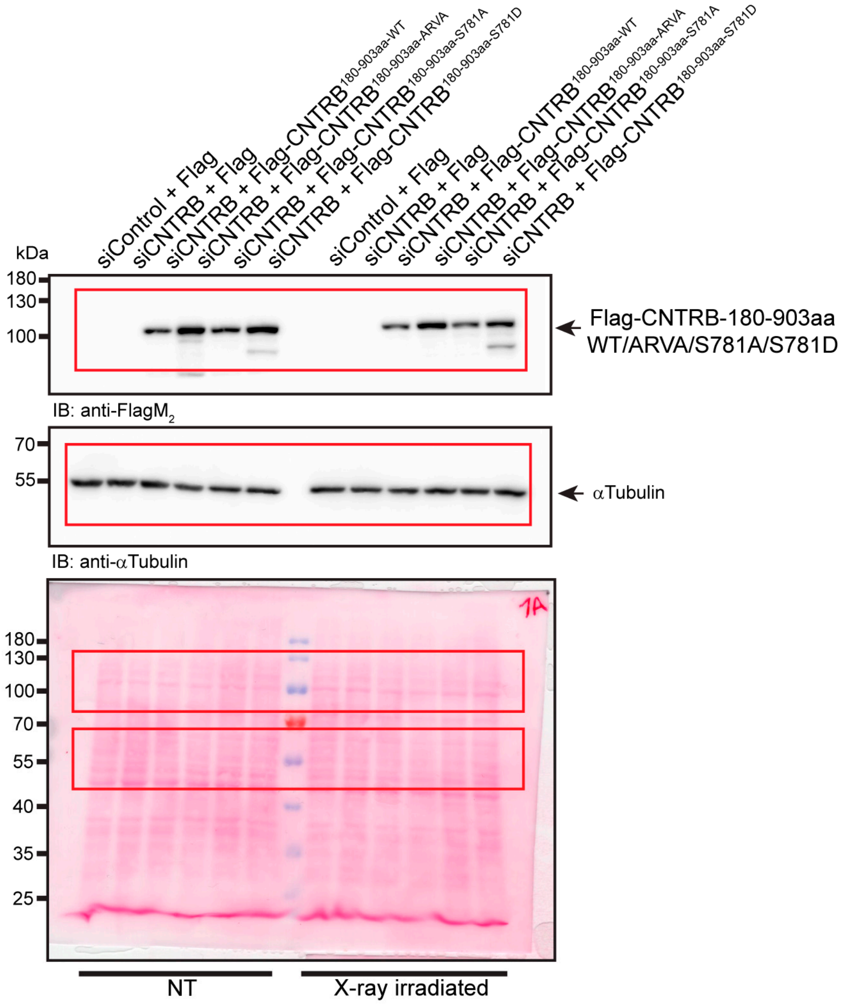

Supplement: Supplementary file 1 [file cells-12-02219-s001.zip › Supplementary Figures.pdf]
